# α‐Adrenergic blockade impairs ductus venosus shunting during an acute period of fetal hypoxaemia without further deficits to cerebral oxygen delivery

**DOI:** 10.1113/EP093085

**Published:** 2026-02-16

**Authors:** Jack R. T. Darby, Stacey L. Holman, Brahmdeep S. Saini, Sunthara Rajan Perumal, Mike Seed, Christopher K. Macgowan, Janna L. Morrison

**Affiliations:** ^1^ Early Origins of Adult Health Research Group, Robinson Research Institute, School of Pharmacy and Biomedical Sciences, College of Health Adelaide University Adelaide South Australia Australia; ^2^ Univeristy of Toronto and The Hospital for Sick Children Toronto Ontario Canada; ^3^ South Australian Health & Medical Research Institute, Preclinical Imaging & Research Laboratories Adelaide South Australia Australia

**Keywords:** α‐adrenergic, ductus venosus, fetus, hypoxaemia, hypoxia, magnetic resonance imaging, oxygen delivery, T_2_ oximetry

## Abstract

The fetal circulation has unique shunts, including the ductus venosus (DV), the tone of which dictates how much substrate‐rich blood returning from the placenta is streamed preferentially away from the liver and instead towards the heart. Herein, we aimed to use clinically relevant MRI techniques in sheep to measure indirectly induced changes in DV tone and the consequences of this on DV shunting, expressed as a ratio of umbilical vein (UV) flow [DV:UV (as a percentage)], and its impact on oxygen delivery to the fetal brain. At 116–117 days gestational age (term = 150 days), fetal sheep (*n* = 11) underwent surgery to implant vascular catheters. Fetal MRI scans were performed at 120–123 days gestational age. Phase contrast MRI and T_2_ MRI oximetry were performed to measure blood flow and oxygen saturation within the fetal circulation during states of fetal normoxaemia, hypoxaemia and hypoxaemia + systemic α‐adrenergic blockade (fetal infusion of the α‐adrenergic antagonist, phentolamine). Hypoxaemia reduced both overall fetal and cerebral oxygen delivery but did not impact either systemic or cerebral oxygen consumption. Fetal hypoxaemia alone did not impact DV shunting. However, addition of α‐adrenergic blockade to acute hypoxaemia significantly decreased DV shunting. Neither fetal hypoxaemia nor the addition of the α‐adrenergic blockade impacted left ventricular cardiac output or blood flow to the brain and the placenta. At the gestational age studied, systemic α‐adrenergic signalling is required to maintain DV shunting during fetal hypoxaemia. However, reduced DV shunting during the systemic α‐adrenergic blockade was not accompanied by a further deficit in cerebral oxygen delivery_._

## Abstract (highlights)

- **What is the central question of this study?**
  Ductus venosus (DV) tone is dynamic, in that it can dilate or constrict to allow more or less blood to be streamed preferentially towards the left heart and brain. We set out to use clinically relevant MRI techniques in sheep to confirm that indirect manipulation of DV tone via α‐adrenergic blockade is possible and determine the impact of this on cerebral oxygen delivery.
- **What is the main finding and its importance?**
  Without increased DV shunting, cerebral oxygen delivery falls during periods of acute hypoxaemia. Delineating the relationship between the extent of DV shunting and preservation of cerebral oxygen delivery might provide valuable insight during assessment of at‐risk fetuses.

## 1 INTRODUCTION

Unlike life after birth, where the gaseous exchange of oxygen and carbon dioxide occurs in the lungs, before birth the responsibility of ensuring not only appropriate gaseous exchange but also nutrient transfer from the maternal to the fetal circulation falls to the placenta. To account for this differing origin of oxygen‐ and nutrient‐rich blood, the fetal circulation includes unique shunts, namely, the ductus venosus (DV), the foramen ovale (FO) and the ductus arteriosus (DA). These shunts close after birth to allow an efficient transition of gaseous exchange from the placenta to the lungs (Holmes et al., 2024). However, during fetal life these three shunts work in tandem to ensure that appropriate amounts of oxygen and nutrients are delivered to vital organs, such as the heart and brain, by reducing the delivery of these substrates to the liver and lungs (Rudolph & Heymann, 1967).

When oxygen‐ and nutrient‐rich blood returns from the placenta to the fetus via the umbilical vein (UV), it either perfuses the liver, becoming less substrate rich along the way, or it maintains its substrate content by passing directly into the inferior vena cava by way of the DV. Spiralling along the posterior side of the inferior vena cava, this substrate‐rich blood is then preferentially streamed towards the left side of the heart via the FO, then towards the brain (Edelstone & Rudolph, 1979; Schmidt et al., 1996; Schrauben et al., 2019). Blood entering the right side of the heart that does not pass through the FO makes up right ventricular cardiac output, which, in order not to perfuse the fluid‐filled lungs unnecessarily, is largely funnelled through the DA and into the descending aorta (Mielke & Benda, 2001; Prsa et al., 2014; Rudolph, 1985). Indeed, studies in fetal sheep have shown that only 7%–8% of combined ventricular output is directed towards the lungs in utero, which is regulated predominately via the tone of the pulmonary vasculature (Holmes et al., 2024; Rudolph, 1979). Altering the tone of these aforementioned shunts has the direct and significant potential to influence blood flow and substrate delivery throughout the fetal circulation, including its distribution towards the fetal brain; a feat that might prove useful in the setting of fetal hypoxaemia, when cerebral oxygen delivery ($D_{O_{2}}$) is impaired (Darby et al., 2024). However, the precise mechanisms that regulate the tone of these shunts is still not fully elucidated, hence a clinical target is currently out of reach.

The patency of the FO is thought to be driven entirely by the pressure gradient across the right and left sides of the fetal heart, whereas the tone of both the DA and DV is responsive to vasoactive agents (Adeagbo et al., 1982; Poon, 2007; Rudolph, 1983; Thomas et al., 2005; Zielinsky & Busato, 2013; Zielinsky et al., 2013). For example, a drop in circulating prostaglandin concentrations at the time of birth when the placental circulation is removed results in the normal constriction, then closure of the DA. This response is replicated with the use of ibuprofen or indomethacin to block prostaglandin synthesis when the DA remains patent after birth and the reason why their use during pregnancy is contraindicated (Ohlsson et al., 2015; Poon, 2007). In contrast, inhibition of endogenous prostaglandin synthesis has no impact on DV tone (Paulick et al., 1990a). However, addition of exogenous prostaglandin I_2_ dilates the DV in vitro when measured by wire myography, and more recently, we have used four‐dimensional flow MRI for in vivo confirmation (Adeagbo et al., 1985; Darby et al., 2020). It should be noted that with respect to the DV, much more is known about the indirect mechanisms that govern its tone.

The DV dilates in response to fetal hypoxaemia, and we and others have shown increased DV shunting during both fetal growth restriction (Bellotti et al., 1998, 2004; Darby et al., 2024; Kiserud et al., 2006) and episodes of acute hypoxia (Behrman et al., 1970; Kiserud et al., 2000; Paulick et al., 1991a). Importantly, this is not a consequence of the DV responding directly to the lower oxygen tension but rather attributable to an α‐adrenergic receptor‐induced vasoconstriction of the surrounding vasculature. For example, not only have we shown previously that chronically hypoxaemic growth‐restricted fetal sheep are hyperinnervated at the level of the periphery and more reliant on α‐adrenergic signalling to regulate the blood pressure, but that they also have increased streaming through the DV–FO pathway, indicative of DV dilatation (Danielson et al., 2005; Darby et al., 2021, 2024). Moreover, seminal work in fetal sheep found a similar response when the hypoxaemia was more acute in nature (Paulick et al., 1990b) and, importantly, that this could be replicated with a fetal infusion of catecholamines (Paulick et al., 1991b). Building on this, the same researchers showed that increased DV shunting in response to acute hypoxaemia was at the expense of hepatic blood flow, which was inhibited by α‐adrenergic blockade (Paulick et al., 1991a). However, although their study reported the impact of hypoxaemia and hypoxaemia + α‐adrenergic blockade on blood flows to the liver, adrenals and kidneys, they were unable to report blood flow and/or $D_{O_{2}}$ to the fetal heart and brain. As such, a conclusion on whether manipulation of DV tone via the α‐adrenergic system would impact cerebral $D_{O_{2}}$ could not be drawn.

Herein, we aimed to use clinically relevant MRI techniques to confirm that indirect manipulation of DV tone via α‐adrenergic blockade is possible and to build on this by also measuring blood flow and $D_{O_{2}}$ to the fetal brain. We hypothesized that acute hypoxaemia would increase DV shunting, resulting in a maintenance of cerebral $D_{O_{2}}$, but that superimposing an α‐adrenergic blockade on the prevailing level of fetal hypoxaemia would mitigate this response and thus reduce cerebral $D_{O_{2}}$.

## 2 MATERIALS AND METHODS

### 2.1 Ethical approval

All experimental protocols were reviewed and approved by the Animal Ethics Committee of the South Australian Health and Medical Research Institute (SAHMRI; ethics approval number, SAM 389.19) and abide by the Australian Code of Practice for the Care and Use of Animals for Scientific Purposes developed by the National Health and Medical Research Council. Ewes from the SAHMRI farm (Burra, South Australia) were housed in an indoor facility with a constant ambient temperature of 20°C–22°C and a 12 h–12 h light–dark cycle. Ewes were housed in individual pens in view of other sheep and had ad libitum access to food and water. All investigators understood the ethical principles outlined by Grundy (2015) and the principles of the 3Rs, specifically the reduction of the use of animals in research (Russell & Burch, 1959). Reporting requirements for ethics and welfare in *Experimental Physiology* (O'Halloran, 2024) have been followed.

### 2.2 Fetal catheterization surgery

At 116–117 days gestational age (GA; term = 150 days), Merino ewes (*n* = 11) carrying singleton fetuses (male, *n* = 2; female, *n* = 9) underwent surgery in aseptic conditions, as previously described (Edwards et al., 1999; Morrison et al., 2001). General anaesthesia was induced with intravenous diazepam (0.3 mg/kg) and ketamine (5 mg/kg), then maintained with inspired isoflurane (1.5%–2.5% in 100% oxygen). A depth of anaesthesia appropriate for commencement of surgical procedures was determined via absence of the eye reflex. Vascular catheters were filled with heparinized saline (0.9% NaCl) and implanted into the maternal jugular vein, fetal femoral vein, UV, femoral artery and the amniotic cavity as described previously (Darby et al., 2020; Edwards et al., 1999; Morrison et al., 2001). The uterus was then closed in two watertight layers around the catheters, and the catheters were tunnelled through an incision in the flank of the ewes. Catheters were flushed daily with heparinized saline (0.9% NaCl). Ewes received an intramuscular injection of antibiotics [3.5 mL of Duplocillin (150 mg/mL procaine penicillin and 112.5 mg/mL benzathine penicillin; Norbrook Laboratories Ltd, Gisborne, VIC, Australia)] and 2 mL of 125 mg/mL Dihydrostreptomycin (Sigma‐Aldrich, St Louis, MO, USA) at surgery and daily for 3 days following surgery. Fetuses received an intramuscular injection of 1 mL of Duplocillin (150 mg/mL procaine penicillin and 112.5 mg/mL benzathine penicillin) and 1 mL of 125 mg/mL dihydrostreptomycin during surgery. All ewes received an analgesic, meloxicam (0.5 mg/kg, subcutaneously) on the day before surgery and 24 h later (Varcoe et al., 2019). Each fetus received antibiotics (500 mg; sodium ampicillin, Commonwealth Serum Laboratories, Melbourne, VIC, Australia) intra‐amniotically for 4 days postsurgery.

### 2.3 Experimental protocol

Pregnant ewes underwent MRI scans between 120 and 123 days GA after ∼16 h of fasting. General anaesthesia was induced in the ewe as described for surgery above. The ewe was intubated, then positioned on its left side for the duration of the scan and ventilated to ensure normal fetal oxygenation levels (respiratory rate 16–18 breaths/min; ∼2 L O_2_ and 4 L air). Maternal heart rate and arterial oxygen saturation ($S_{aO_{2}}$) were measured using an MRI‐compatible $S_{aO_{2}}$/heart rate monitor (Nonin Medical Inc., Plymouth, MN, USA) and maternal temperature were measured with an MRI‐safe fibre‐optic temperature probe (OSENSA Innovations Corp., Burnaby, BC, Canada). The $S_{aO_{2}}$/heart rate sensor was placed on a teat of the pregnant ewe and the temperature probe placed in the rectum of the ewe, with measurements continuously recorded using LabChart 7 (Darby et al., 2019; Duan et al., 2019). The depth of maternal anaesthesia was assessed as a combination maternal eye reflex, sudden changes in maternal heart rate and/or the presence of rebreathing.

The fetal femoral artery and amniotic catheters were connected to displacement transducers, a quad‐bridge amplifier and a data acquisition unit (PowerLab, ADInstruments, Castle Hill, NSW, Australia) to record fetal blood pressure (corrected for amniotic pressure). All data were sampled at a rate of 1000 Hz, digitized and recorded using LabChart 7 (ADInstruments). The resulting blood pressure signal acted as a real‐time external cardiac trigger for fetal MRI scanning (Duan et al., 2017, 2019; Schrauben et al., 2019).

Imaging was performed on a 3 T clinical MRI system (MAGNETOM Skyra, Siemens Healthineers, Erlangen, Germany). Fetal blood flow measurements and oxygen saturations were determined by phase contrast (PC)‐MRI and T_2_ MRI oximetry as described previously (Darby et al., 2020; Duan et al., 2019; Saini et al., 2020). MRI measurements were taken initially in a basal state of fetal normoxaemia, then during a period of fetal hypoxaemia, and then during a period of fetal hypoxaemia with a continuous intravenous infusion of the α‐adrenergic antagonist, phentolamine (5 mg bolus + 0.25 mg/min infusion; Sigma‐Aldrich) into the UV (Danielson et al., 2005; Darby et al., 2021). Fetal hypoxaemia was achieved by reducing maternal inspired oxygen to room air (21%) and, if necessary, offsetting the oxygen percentage of the inspired gas with the addition of nitrogen to reach a target fetal partial pressure of oxygen ($P_{aO_{2}}$) of 12–13 mmHg. MRI acquisition during the fetal hypoxaemia and fetal hypoxaemia + phentolamine states began 10 min after the establishment of fetal hypoxaemia or after the beginning of the phentolamine infusion, respectively. The MRI acquisitions for each state took ∼45–60 min.

### 2.4 Determination of blood flow within the fetal circulation

The fetal femoral arterial pressure waveform was used to generate a cardiac trigger for MRI (Duan et al., 2017; Schrauben et al., 2019). Two‐dimensional cine PC imaging was performed to measure blood flow within the fetal circulation, with corresponding vessel‐appropriate velocity encoding (VENC). PC‐MRI acquisitions were completed for the ascending aorta (AAo; 150 cm/s), descending aorta (DAo; 150 cm/s), superior vena cava (SVC; 100 cm/s), combined carotid arteries (CCa; 100 cm/s), umbilical vein (UV; 50 cm/s) and ductus venosus (DV; 100 cm/s) using the following parameters: flip angle = 30°; repetition time = 7 ms; echo time = 3.18 ms; field of view = 240 mm; in‐plane resolution = 1.0 mm × 1.0 mm; slice thickness = 5.0 mm; number of signal averages = 3; and views per segment = 2, according to our previously published technique (Cho et al., 2020; Dimasi et al., 2021; Duan et al., 2019). With 15 acquired phases in the cardiac cycle, these parameters achieve a temporal resolution of ∼30 ms. The typical acquisition time for each vessel was ∼2 min. PC cine images were acquired in the short‐axis plane of the vessels of interest, which were prescribed using two perpendicular long‐axis views of each vessel. PC acquisitions of each fetal vessel were analysed using the freely available software Segment v.4.0 R12067 [Medviso, segment.heiberg.se; (Heiberg et al., 2010)]. Left ventricular output was determined as equal to AAo blood flow and did not include coronary blood flow. Blood flow towards the lower trunk of the fetus was determined as DAo less the blood flow to the umbilical arteries (deemed equal to UV blood flow).

### 2.5 Determination of oxygen saturation within the fetal circulation

Owing to the paramagnetic properties of deoxyhaemoglobin, the T_2_ relaxation time of blood is related to the oxygen saturation of blood (Christen et al., 2013). Vessel T_2_ oximetry was performed using a T_2_‐prepared pulse sequence with a balanced steady‐state free precession acquisition (Myomaps, Siemens) (Saini et al., 2020; Sun et al., 2015; Xu et al., 2020; Zhu et al., 2016). In‐plane resolution was 1.3 mm × 1.3 mm. MRI acquisition parameters over all subjects and vessels were: repetition time = 4.2 ms, echo time = 2.1 ms, flip angle = 70°, slice thickness = 6 mm, T_2_ preparation times = [32, 64, 96, 128, 160, 192] ms, and acquisition time ≈ 50 s. A non‐rigid motion‐correction algorithm (co‐registration) was applied to compensate for slight in‐plane fetal movement (Giri et al., 2012).

The T_2_ relaxation time for each vessel of interest was analysed using Segment v.4.0 R12067 (Medviso, segment.heiberg.se; (Heiberg et al., 2010)). The regions of interest were adjusted manually for each image slice to cover the central 60% of the vessel of interest (UV, AAo, DAo and SVC) (Stainsby & Wright, 1998). Oxygen saturation was then calculated from T_2_ relaxation time using the T_2_–oxygen saturation relationship for sheep blood as described previously (Saini et al., 2020).

### 2.6 Determination of $D_{O_{2}}$ and oxygen consumption

Blood flow and T_2_‐derived oxygen saturations were combined to calculate overall fetal $D_{O_{2}}$, fetal oxygen consumption (${\dot{V}}_{O_{2}}$), cerebral $D_{O_{2}}$ and cerebral ${\dot{V}}_{O_{2}}$ using the following equations:

$$\mathrm{Fetal}\;D_{O_{2}}=1.36\times \left[\mathrm{Hb}\right]\;\times Y_{\mathrm{UV}}\times Q_{\mathrm{UV}}$$

$$\mathrm{Cerebral}=1.36\times \left[\mathrm{Hb}\right]\times Y_{\mathrm{AAo}}\times Q_{\mathrm{CCa}}$$

$$\mathrm{Fetal}\;=1.36\times \left[\mathrm{Hb}\right]\times \left(Y_{\mathrm{UV}\;}-Y_{\mathrm{DAo}}\right)\;\times Q_{\mathrm{UV}}$$

$$\mathrm{Cerebral}\;=1.36\times \left[\mathrm{Hb}\right]\times \left(Y_{\mathrm{AAo}\;}-Y_{\mathrm{SVC}}\right)\;\times Q_{\mathrm{CCa}}$$

where: *Q*_UV_ represents the measured UV blood flow; *Q*_CCa_ represents the combined blood flow of the carotid arteries; [Hb] represents the mean fetal haemoglobin concentration during the MRI scan; 1.36 is the amount of oxygen (in millilitres at 1 atm) bound per gram of haemoglobin; *Y*_UV_ represents the oxygen saturation of UV blood; *Y*_DAo_ represents the oxygen saturation of the DAo blood; *Y*_AAo_ represents the oxygen saturation of AAo blood; and *Y*_SVC_ represents the oxygen saturation of the SVC.

### 2.7 Blood sampling and fetal blood gas measurements

Fetal arterial blood samples (0.5 mL) were collected daily to monitor fetal health by measuring the $P_{aO_{2}}$, partial pressure of carbon dioxide ($P_{\mathrm{aC}O_{2}}$), oxygen saturation ($S_{aO_{2}}$), pH, haemoglobin (Hb), haematocrit (Hct), base excess and lactate concentrations, temperature corrected to 39°C for sheep blood with a RAPIDPOINT 500 (Siemens Healthineers, Melbourne, VIC, Australia). During the MRI scan, arterial samples (0.5 mL) for fetal blood gas analysis were taken at the beginning, middle and end of each of the three states, then averaged to reflect fetal blood gas status during each state.

### 2.8 Post‐mortem examination

At 123–124 days GA, pregnant ewes were humanely killed with an intravenous overdose of sodium pentobarbitone (150 mg/kg body weight; Virbac, Peakhurst, NSW, Australia) and death confirmed by the absence of both maternal eye reflex and venous return. The fetus was delivered via hysterotomy and weighed. The fetal body and brain were weighed for blood flow normalization.

### 2.9 Statistical analysis

To determine the effect of fetal hypoxaemia and fetal hypoxaemia + α‐adrenergic blockade on fetal haemodynamics, a mixed‐effect model (random effect accounting for repeated measures of a specific fetus across MRI states) was used, followed by Bonferroni's correction for multiple comparisons (GraphPad Prism v.8, GraphPad Software, La Jolla, CA, USA). Data are presented as the mean ± SD, and a probability of 5% (*P *< 0.05) was considered significant for all analyses. During the study, three fetal sheep died either during the α‐adrenergic blockade or immediately after the completion of the α‐adrenergic blockade acquisitions occurred. For transparency, these fetuses are represented as triangles instead of circles in the figures.

## 3 RESULTS

### 3.1 Fetal blood gas, pH, Hb, Hct and lactate measures

Fetal $P_{aO_{2}}$ (*P *< 0.0001) and $S_{aO_{2}}$ (*P *< 0.0001) were significantly lower during the hypoxaemia and hypoxaemia + α‐adrenergic blockade state than the normoxaemia (Table 1). However, fetal $P_{aO_{2}}$ and $S_{aO_{2}}$ remained similar during the hypoxaemia state and with the addition of the α‐adrenergic blockade. Fetal lactate concentrations were significantly higher in the hypoxaemia state than the normoxaemia state and higher still with the addition of the α‐adrenergic blockade (*P* = 0.0014; Table 1). Fetal $P_{\mathrm{aC}O_{2}}$ (*P* = 0.0907), pH (*P* = 0.3487), Hb (*P* = 0.0564) and Hct (*P* = 0.0553) remained stable throughout the MRI session (Table 1).

**TABLE 1:** Fetal blood gases, haemoglobin, haematocrit and lactate values prior to anaesthesia for MRI and during normoxaemia, hypoxaemia and hypoxaemia + α‐adrenergic blockade MRI acquisition states.

Parameter	Normoxaemia state (*n* = 11)	Hypoxaemia state (*n* = 11)	Hypoxaemia *+* α‐adrenergic blockade (*n* = 11)	*P*‐value
$P_{aO_{2}}$, mmHg	21.2 ± 1.8^a^	12.9 ± 1.5^b^	12.3 ± 1.2^b^	<0.0001
$P_{\mathrm{aC}O_{2}}$, mmHg	55.6 ± 6.3	50.4 ± 5.6	50.4 ± 6.5	0.0907
pH	7.291 ± 0.026	7.303 ± 0.047	7.291 ± 0.055	0.3487
$S_{aO_{2}}$, %	58.4 ± 6.1^a^	28.5 ± 6.0^b^	25.5 ± 3.7^b^	<0.0001
Haemoglobin, g/L	93 ± 7	91 ± 8	89 ± 7	0.0564
Haematocrit, %	27 ± 2	27 ± 2	26 ± 2	0.0553
Lactate, mmol/L	1.82 ± 0.46^a^	3.18 ± 1.82^b^	4.68 ± 2.41^c^	0.0014
Maternal temperature, °C	39.29 ± 0.39	39.31 ± 0.41	39.38 ± 0.49	0.3370

*Note*: Values are the mean ± SD. Ewes were anaesthetized and lying on their left side during MRI. Data were analysed by a mixed‐effects model with Bonferroni's correction for multiple comparisons. Superscript alphabetical letters indicate significant differences between states (*P *< 0.05) such that values with different alphabetical letters are statistically different from each other and values with the same alphabetical letters are not different. Abbreviations: $P_{\mathrm{aC}O_{2}}$, arterial partial pressure of carbon dioxide; $P_{aO_{2}}$, arterial partial pressure of oxygen; $S_{aO_{2}}$, arterial oxygen saturation.

### 3.2 Impact of hypoxaemia and α‐adrenergic blockade on fetal blood pressure and heart rate

There was no difference in fetal systolic (*P* = 0.1290), diastolic (*P* = 0.2336) or mean (*P* = 0.1168) arterial pressures between normoxaemia, hypoxaemia and hypoxaemia + α‐adrenergic blockade MRI acquisition states (Table 2). Fetal heart rate was not different between normoxaemia and hypoxaemia states. However, fetal heart rate was significantly higher during the hypoxaemia + α‐adrenergic blockade state than during both the normoxaemia and hypoxaemia states (*P* = 0.0110; Table 2).

**TABLE 2:** Impact of hypoxaemia and α‐adrenergic blockade on fetal blood pressure and heart rate during the MRI.

Parameter	Normoxaemia state (*n* = 11)	Hypoxaemia state (*n* = 11)	Hypoxaemia + α‐adrenergic blockade (*n* = 11)	*P*‐value
SBP, mmHg	50 ± 10	53 ± 12	48 ± 10	0.1290
DBP, mmHg	29 ± 10	31 ± 11	29 ± 10	0.2336
MAP, mmHg	37 ± 9	40 ± 12	37 ± 10	0.1168
Heart rate, beats/min	148 ± 13^a^	163 ± 15^a^	185 ± 34^b^	**0.0110**

*Note*: Values are the mean ± SD. Data were analysed by a repeated‐measures one‐way ANOVA with Bonferroni's correction for multiple comparisons. Superscript alphabetical letters indicate significant differences between states (*P *< 0.05) such that values with different alphabetical letters are statistically different from each other and values with the same alphabetical letters are not different. Abbreviations: DBP, diastolic blood pressure; MBP, mean blood pressure; SBP, systolic blood pressure.

### 3.3 Impact of hypoxaemia and α‐adrenergic blockade on DV shunting

**FIGURE 1:**
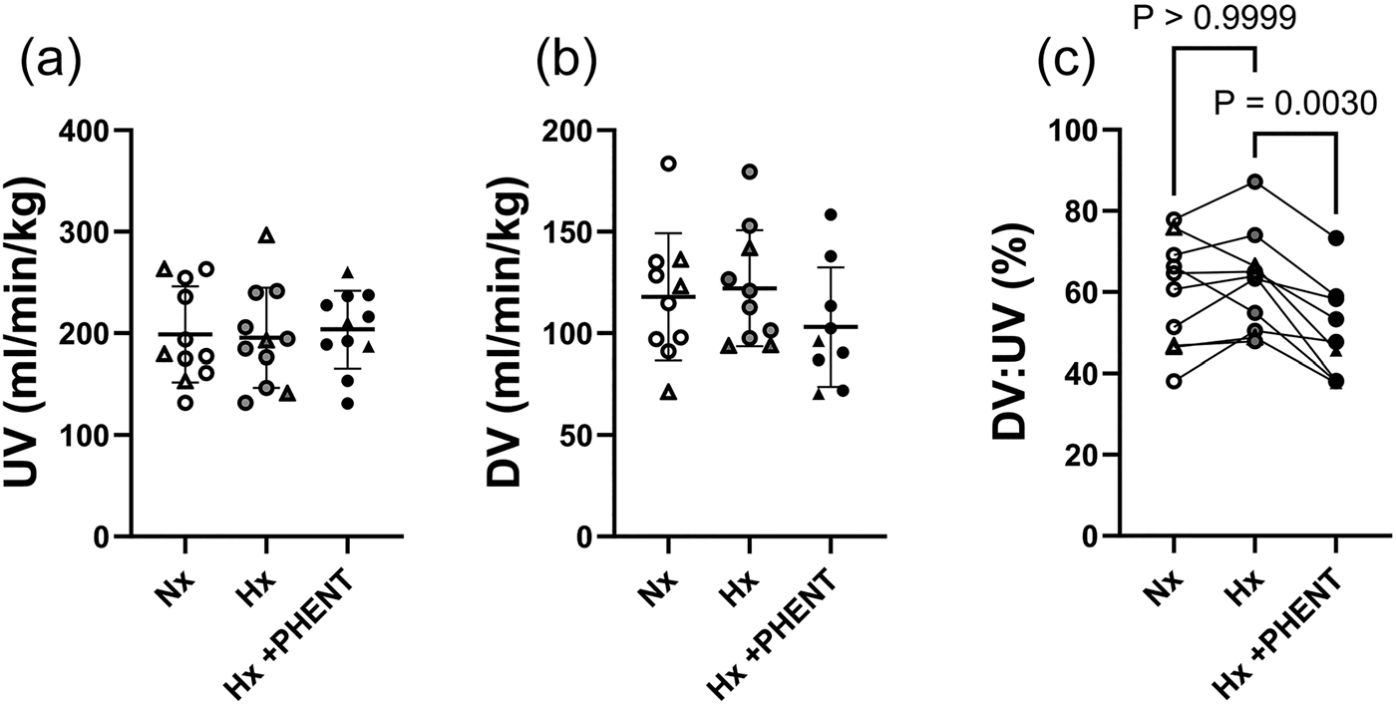
Blood flow through the umbilical vein (a), ductus venosus (b) and ductus venosus shunting (c) during normoxaemia (unfilled symbols), hypoxaemia (grey filled symbols) and α‐adrenergic blockade (black filled symbols) MRI acquisition windows. Triangles represent those fetuses that died either during the α‐adrenergic blockade state or in the immediate aftermath of the MRI acquisition. The MRI image for DV was impacted by artefact and not analysable for one fetus in each of the Nx, Hx and Hx + PHENT states. Data are presented as individual points, with the mean ± SD superimposed. Data were analysed by a mixed‐effect model with Bonferroni's correction for multiple comparisons (*P* ≤ 0.05). Abbreviations: DV, ductus venosus; Hx, hypoxaemia; Hx + PHENT, hypoxaemia + phentolamine; Nx, normoxaemia; UV, umbilical vein.

There was no impact of hypoxaemia or α‐adrenergic blockade on absolute blood flow through the UV (Figure 1a) or the DV (Figure 1b). There was no impact of hypoxaemia on DV shunting (DV:UV flow ratio); however, addition of α‐adrenergic blockade significantly reduced DV shunting in comparison to the hypoxaemia but not the normoxaemia state (Figure 1c; *P* = 0.0030).

### 3.4 Impact of hypoxaemia and α‐adrenergic blockade on blood flow within the fetal circulation

**FIGURE 2:**
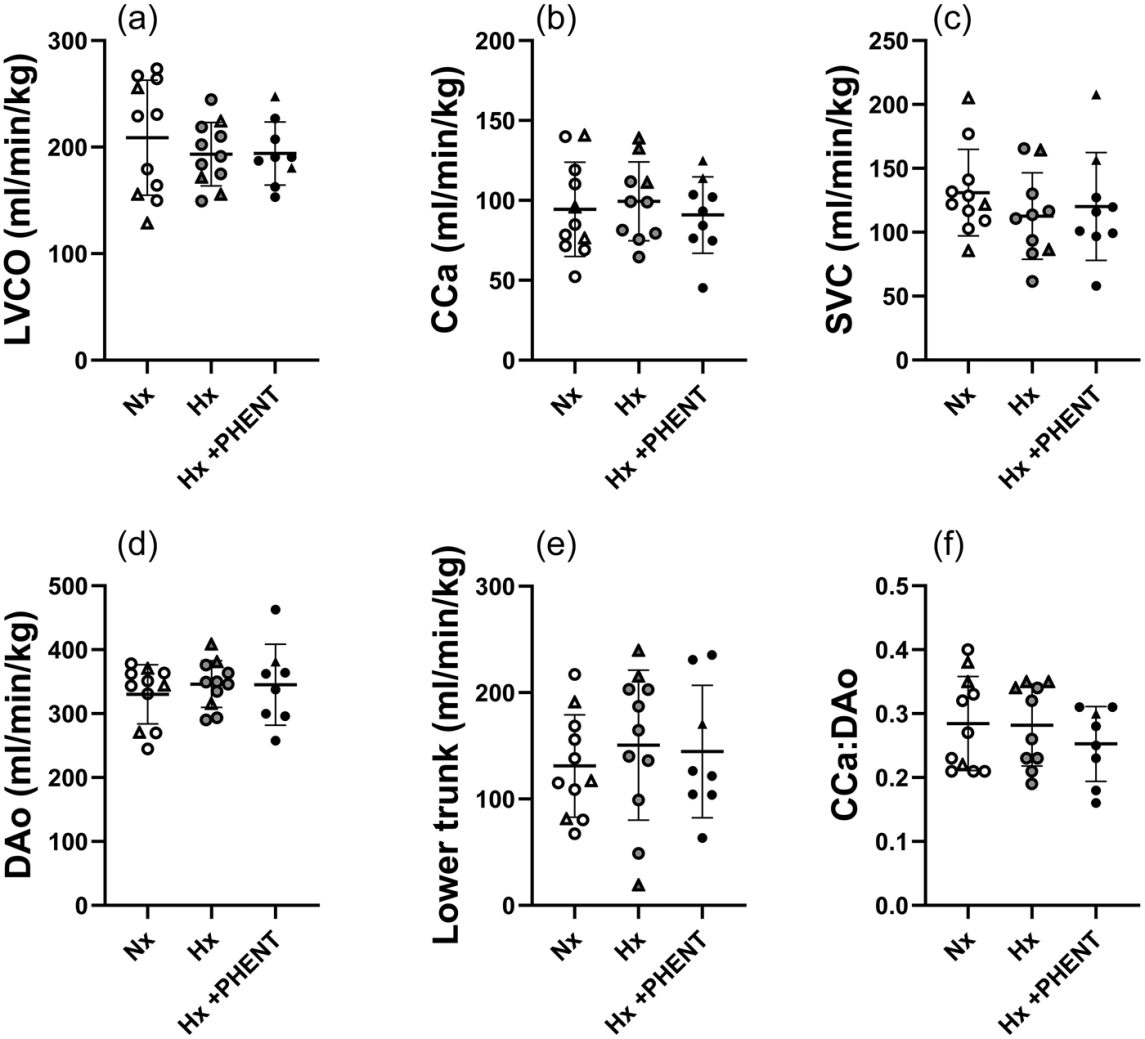
Fetal LVCO (a), CCa blood flow (b), blood flow through the SVC (c), blood flow through the DAo (d), blood flow towards the lower trunk of the fetus (e) and the ratio of CCa:DAo blood flow (pseudo‐measure of brain sparing physiology; f) during normoxaemia (unfilled symbols), hypoxaemia (grey filled symbols) and α‐adrenergic blockade (black filled symbols) MRI acquisition windows. Triangles represent those fetuses that died either during the α‐adrenergic blockade state or in the immediate aftermath of the MRI acquisition. The PC‐MRI image for SVC was not appropriate for analysis in one fetus during the Hx state. The MRI image for CCa was not appropriate for analysis in one fetus during Nx state and one fetus during Hx + PHENT state. The PC‐MRI image for DAo was not appropriate for analysis in one fetus during the Hx + PHENT state. Data are presented as individual points, with the mean ± SD superimposed. Data were analysed by mixed‐effect model with Bonferroni's correction for multiple comparisons (*P* ≤ 0.05). Abbreviations: CCa, combined carotid artery; DAo, descending aorta; Hx, hypoxaemia; Hx + PHENT, hypoxaemia + phentolamine; Nx, normoxaemia; LVCO, left ventricular cardiac output; PC‐MRI, phase contrast‐MRI; SVC, superior vena cava.

Left ventricular output (Figure 2a) and blood flow through the CCa (Figure 2b), SVC (Figure 2c), DAo (Figure 2d) and towards the lower trunk (Figure 2e) were not impacted by hypoxemia or the addition of the α‐adrenergic blockade. The CCa:DAo blood flow ratio (marker of brain‐sparing physiology) remained consistent across all three MRI acquisition states (Figure 2f).

### 3.5 Impact of hypoxaemia and α‐adrenergic blockade on $D_{O_{2}}$ and ${\dot{V}}_{O_{2}}$

**FIGURE 3:**
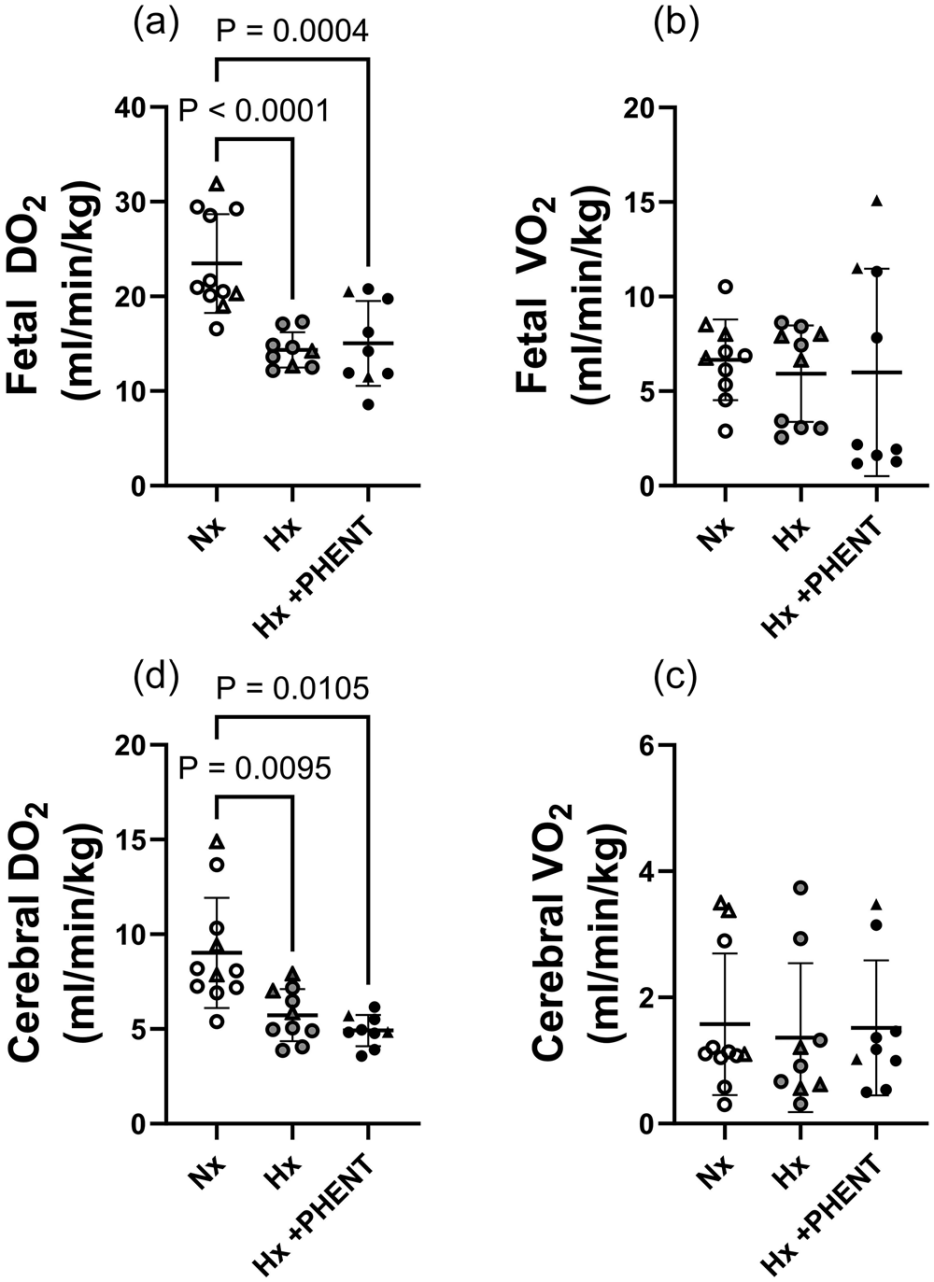
Fetal $D_{O_{2}}$ (a), fetal ${\dot{V}}_{O_{2}}$ (b), cerebral $D_{O_{2}}$ (c) and cerebral ${\dot{V}}_{O_{2}}$ (d) during normoxaemia (unfilled symbols), hypoxaemia (grey filled symbols) and α‐adrenergic blockade (black filled symbols) MRI acquisition windows. Triangles represent those fetuses that died either during the α‐adrenergic blockade state or in the immediate aftermath of the MRI acquisition. The umbilical vein T_2_ MRI image was not appropriate for analysis in one fetus during the Hx state and in one fetus during the Hx + PHENT state. The phase contrast‐MRI image for the combined carotid artery was not appropriate for analysis in one fetus during the Nx state and one fetus during the Hx + PHENT state. Data are presented as individual points with mean ± SD superimposed. Data were analysed by mixed‐effect model with Bonferroni's correction for multiple comparisons (*P* ≤ 0.05). Abbreviations: $D_{O_{2}}$, oxygen delivery; Hx, hypoxaemia; Hx + PHENT, hypoxaemia + phentolamine; Nx, normoxaemia; ${\dot{V}}_{O_{2}}$, oxygen consumption.

Fetal $D_{O_{2}}$ was significantly lower in the hypoxaemia (*P *< 0.001) and hypoxaemia + α‐adrenergic blockade (*P* = 0.004) states in comparison to the normoxaemia state (Figure 3a). There was no significant difference in fetal $D_{O_{2}}$ between the hypoxaemia and hypoxaemia + α‐adrenergic blockade states. Fetal ${\dot{V}}_{O_{2}}$ remained stable across all three states (Figure 3b).

Cerebral $D_{O_{2}}$ was significantly lower in the hypoxaemia (*P* = 0.0095) and hypoxaemia + α‐adrenergic blockade (*P* = 0.0105) states in comparison to the normoxaemia state (Figure 3c). There was no significant difference in cerebral $D_{O_{2}}$ between the hypoxaemia and hypoxaemia + α‐adrenergic blockade states. Cerebral ${\dot{V}}_{O_{2}}$ remained stable across all three states (Figure 3d).

## 4 DISCUSSION

The indirect regulation of DV tone via increased resistance of the hepatic vasculature is generally considered a key physiological mechanism to ensure that even during periods of hypoxaemia an adequate amount of oxygen is delivered to the most vital organs. Dilatation of the DV allows more blood returning from the placenta to be streamed preferentially towards the heart and brain. Much of our knowledge surrounding this process stems from the seminal work in fetal sheep using radioactively labelled microspheres, a technique that is not translatable to the clinic (Rudolph, 1979, 1983, 1985; Rudolph & Heymann, 1967). Herein, we hypothesized that our previously validated and clinically translatable MRI techniques (Darby et al., 2023, 2024; Duan et al., 2019; Saini et al., 2020, 2023; Sun et al., 2021) would be capable of measuring this well‐characterized fetal haemodynamic response to acute hypoxaemia, in addition to the consequences of impairing DV shunting during this response on cerebral $D_{O_{2}}$.

At its core, the present study shares a similar experimental design to previous work investigating this same physiological process (Paulick et al., 1991a). Both studies assessed fetal haemodynamics during a basal state of fetal normoxaemia, a state of fetal hypoxaemia (similar severity; $P_{aO_{2}}$ = 11.7 ± 0.9 mmHg), followed by addition of a systemic α‐adrenergic blockade upon the prevailing level of hypoxaemia. The key methodological differences are our use of PC‐MRI to measure fetal blood flow instead of the prior use of radioactively labelled microspheres and our ability to determine the oxygenation of blood non‐invasively within a given fetal vessel using T_2_ oximetry (Duan et al., 2019; Heymann et al., 1977; Saini et al., 2020). Despite multiple studies, including Paulick et al. (1991a), reporting an increase in DV shunting during acute fetal hypoxaemia and this being a relatively well agreed upon adaptation of the fetal circulation to hypoxaemia (Tchirikov et al., 1998, 2006), we found that hypoxaemia alone did not increase DV shunting. In an effort to increase the surface area of fetal placental tissue and thus improve gaseous exchange during hypoxaemia, increased DV shunting has previously been associated with increased UV resistance and/or decreased UV blood flow (Paulick et al., 1990b; Tchirikov et al., 1998). In the present study, we were unable to measure the vascular resistance of the UV; however, we found no change in UV blood flow. This suggests that should DV shunting have increased in response to acute hypoxaemia, it would have been driven entirely by increased DV flow attributable to elevated hepatic vascular resistance. Although we did not measure hepatic vascular resistance directly, the decreased DV shunting alongside a maintenance of UV blood flow during the α‐adrenergic blockade suggests that hepatic vascular tone still has a degree of influence during hypoxaemia, albeit not to the extent that would be required to increase DV shunting. It is possible that this weakened response might be attributable to the maturational state of the cardiovascular system in fetuses at the GA studied herein.

The ability of the fetal cardiovascular system to respond appropriately to hypoxaemia aligns with a maturation of both neural and endocrine mechanisms as gestation progresses (Boddy et al., 1974; Fletcher et al., 2006; Giussani, 2016). As such, the lack of increased DV shunting during the hypoxaemia state, but still a reliance on the α‐adrenergic system to maintain shunting levels, might be attributable to the GA of the fetuses studied. However, it is often hard to compare the contribution of GA definitively to the type and magnitude of the hypoxaemia‐driven cardiovascular response because the majority of the original microsphere studies were performed in fetuses from a large range of GAs. Paulick et al. (1991) report performing catheterization surgery on their fetuses from 125 to 135 days GA, with the microsphere experiments occurring 1–3 days after surgery. This creates a difference in the GA range between their work and the present study of 2–18 days, which is a significant time for maturational differences to occur that might result in the magnitude and stability of the response being different in those fetuses at the younger end of this range from those at the older end (Segar, 1997; Unno et al., 1999; Wassink et al., 2007). For example, slightly older fetuses (125–130 days GA) than those in the present study display only transient bradycardia and femoral vasoconstriction in response to acute hypoxaemia, whereas this response is more persistent and of a greater magnitude in older near‐term fetuses (∼145 days GA; Fletcher et al., 2006). Moreover, the cardiovascular system of chronically hypoxaemic fetuses becomes increasingly more innervated by the α‐adrenergic system as gestation progresses, indicative of a maturational component (Danielson et al., 2005; Darby et al., 2021). Our results are more in line with our previous work in slightly younger fetuses (1–6 days younger;117–119 days GA), in which chronic but not acute hypoxaemia increased DV shunting (Darby et al., 2024), allowing us to suggest that at least from 117–123 days GA the mechanisms responsible for the indirect regulation of DV tone during periods of acute fetal hypoxaemia are not fully mature.

A key aspect of the present study was our endeavour to determine how cerebral $D_{O_{2}}$ was impacted when the hypoxaemia‐driven increased DV shunting was impaired via the α‐adrenergic blockade; a measure not reported previously (Paulick et al., 1991a). However, we did not anticipate that the indirect α‐adrenergic regulation of DV tone would not yet be fully functional. As such, we were unable to measure cerebral $D_{O_{2}}$ whilst the systemic hypoxaemia was driving an increase in DV shunting. Instead, our measure of cerebral $D_{O_{2}}$ was taken during a period of acute fetal hypoxaemia without increased DV shunting. In line with previous work from both our research group and others, we found that cerebral $D_{O_{2}}$ decreased significantly during hypoxaemia (Darby et al., 2024; Newman et al., 2000). Interestingly, older fetuses (128–138 days GA) than those in the present study but within the GA range of those shown to have a fully developed DV shunting response to hypoxaemia still exhibited a decline in cerebral $D_{O_{2}}$ (Rurak et al., 1990). It would, of course, be interesting to know whether there is a direct relationship between the magnitude of rise in DV shunting and the magnitude in fall of cerebral $D_{O_{2}}$ during systemic hypoxaemia. Although it is assumed that there would be a direct relationship, to the best of our knowledge no study has directly defined this. On the other end of the spectrum, when DV shunting fell during the α‐adrenergic blockade, there was no further deficit to cerebral $D_{O_{2}}$. This was unexpected, because a fall in DV shunting suggests a rise in blood flow towards the brain that has already passed through the hepatic circulation and is likely to be more deoxygenated than it otherwise would have been. Indeed, our T_2_ oximetry analysis of blood leaving the AAo showed no change in oxygenation from before to after the α‐adrenergic blockade. We note there are two potential haemodynamic alterations that might explain this. Firstly, although systemic ${\dot{V}}_{O_{2}}$ was unchanged in response to either hypoxaemia or α‐adrenergic blockade, hepatic ${\dot{V}}_{O_{2}}$ might have decreased to prevent a further deficit in cerebral $D_{O_{2}}$. Answering this hypothesis is beyond the scope of the present study; however, future studies could measure hepatic oxygen kinetics comprehensively during periods of fetal hypoxaemia. Secondly, right‐to‐left heart shunting through the FO might have increased during the α‐adrenergic blockade; however, this is unlikely. The well‐characterized response of the pulmonary arteries to hypoxaemia is constriction to increase FO flow. Fetal pulmonary arteries express α‐adrenergic receptors (Shaul et al., 1990), and thus the α‐adrenergic blockade with subsequent pulmonary artery dilatation is more likely to be at the detriment of FO flow. Unfortunately, we were unable to determine FO flow in the present study because it requires a full assessment of pulmonary haemodynamics (Holmes et al., 2024; Morrison et al., 2022).

A key difference of the present study in comparison to the majority of prior studies assessing the impact of hypoxaemia on DV shunting was our use of MRI to measure blood flow and distribution instead of labelled microspheres. It should be acknowledged that, for the most part, those studies using microspheres assessed the immediate response of the DV to hypoxaemia, whereas our use of MRI‐based techniques assessed the haemodynamic response to sustained acute hypoxaemia. Although the MRI techniques used herein are non‐invasive and clinically translatable, a limitation of this study is that the pregnant ewes required anaesthesia during the MRI scans to remain still and unstressed, because sheep are flock animals. To understand the influence of maternal and thus fetal exposure of anaesthesia on haemodynamics, we previously compared the impact that different anaesthetic agents have on the ability of fetuses to respond to a period of acute hypoxaemia, with isoflurane having the least impact on fetal haemodynamics (Varcoe et al., 2020). However, isoflurane has been linked to a vasodilatation of both cerebral and hepatic circulations, with specific alterations in cerebral oxygen kinetics (Gatecel et al., 2003; Matta et al., 1999; McClaine et al., 2005). As such, a prevailing state of altered cerebral and hepatic haemodynamics might have confounded our ability to detect the full extent of alterations in the fetal circulation induced by hypoxaemia and α‐adrenergic blockade. Indeed, it is possible that the lack of increased DV shunting during the hypoxaemia state is not, as we have previously suggested, attributable to the maturational state of the fetal circulation, but rather attributable to the vasodilatory capacity of isoflurane working against the α‐adrenergic receptor‐induced vasoconstriction of the hepatic vasculature. Additionally, given that the DV:UV flows during the normoxaemia state of the present study are higher (mean, 60%; range, 38%–77%) than what has been reported previously in normoxic fetal sheep in the seminal microsphere studies (mean, 53%; range, 36%–64%), there might be less room to increase DV shunting during acute hypoxaemia than if shunting had initially been lower (Edelstone et al., 1978). The causes of this could be teased out in future studies with the use of spinal anaesthesia instead of isoflurane (Davies et al., 2023; Martherus et al., 2020), although from an animal welfare perspective mild maternal sedation would also be required, and thus this approach would not be void of haemodynamic influence.

## 5 CONCLUSION

Overall, we have shown that the well‐characterized DV shunting response to acute hypoxaemia was not present in the fetal sheep at the GA studied, although the decrease in DV shunting during the α‐adrenergic blockade might indicate that some level of α‐adrenergic innervation of the hepatic vasculature was present. Should the lack of increased DV shunting be attributable to the current maturational state of the fetal cardiovascular system, it might suggest that human fetuses at equivalent stages of maturation are at an increased risk for poor outcomes following periods of acute hypoxaemia, such as those caused by umbilical cord occlusions and/or uterine contractions, than older fetuses. We had expected cerebral $D_{O_{2}}$ to worsen with impaired DV shunting. However, the fact that this did not occur suggests either that adaptations have occurred elsewhere in the fetal circulation or that further deficits to cerebral $D_{O_{2}}$ were masked by the vasodilatory effects of anaesthesia. To address these working hypotheses, future studies should aim to perform a thorough assessment of pulmonary and hepatic haemodynamics in similar experimental conditions and perform a comparison of the haemodynamic response against a group of older fetuses.

## AUTHOR CONTRIBUTIONS

Conception or design of the work: Mike Seed, Christopher K. Macgowan and Janna L. Morrison Acquisition or analysis or interpretation of data for the work: Jack R. T. Darby, Stacey L. Holman, Brahmdeep S. Saini, Mike Seed, Christopher K. Macgowan and Janna L. Morrison Drafting the work or revising it critically for important intellectual content: Jack R. T. Darby, Stacey L. Holman, Brahmdeep S. Saini, Mike Seed, Christopher K. Macgowan, Janna L. Morrison. All authors approved the final version of the manuscript and agree to be accountable for all aspects of the work in ensuring that questions related to the accuracy or integrity of any part of the work are appropriately investigated and resolved. All persons designated as authors qualify for authorship, and all those who qualify for authorship are listed.

## CONFLICT OF INTEREST

The authors have no conflicts of interest.

## Data Availability

The data that support the findings of this study are available from the corresponding author upon reasonable request.
